# No evidence of nonlinear effects of predator density, refuge availability, or body size of prey on prey mortality rates

**DOI:** 10.1002/ece3.3183

**Published:** 2017-06-28

**Authors:** Richard M. Simkins, Mark C. Belk

**Affiliations:** ^1^ Department of Biology Brigham Young University Provo UT USA

**Keywords:** linear response, predator density, predator–prey interactions, refuge, size effects, survival

## Abstract

Predator density, refuge availability, and body size of prey can all affect the mortality rate of prey. We assume that more predators will lead to an increase in prey mortality rate, but behavioral interactions between predators and prey, and availability of refuge, may lead to nonlinear effects of increased number of predators on prey mortality rates. We tested for nonlinear effects in prey mortality rates in a mesocosm experiment with different size classes of western mosquitofish (*Gambusia affinis*) as the prey, different numbers of green sunfish (*Lepomis cyanellus*) as the predators, and different levels of refuge. Predator number and size class of prey, but not refuge availability, had significant effects on the mortality rate of prey. Change in mortality rate of prey was linear and equal across the range of predator numbers. Each new predator increased the mortality rate by about 10% overall, and mortality rates were higher for smaller size classes. Predator–prey interactions at the individual level may not scale up to create nonlinearity in prey mortality rates with increasing predator density at the population level.

## 1 INTRODUCTION

The simplest assumption in a predator–prey system is that prey mortality increases in a linear additive way as more predators are introduced into the system. This assumption includes the caveat that there is no individual heterogeneity among predators or prey (i.e., predators are equally effective in capturing prey, and prey are equally vulnerable to capture) and no interactive effects among predators. However, recent studies have suggested that heterogeneity among predators and predator–predator interactions may generate nonlinear responses in mortality of prey with increasing predator density (Delong & Vasseur, 2013; Pruitt et al., 2017; Stallings & Dingeldein, 2012). Predators can interact with each other as well as with prey to influence mortality among prey via either interference competition or cooperative predation (Delong & Vasseur, 2013; Stallings & Dingeldein, 2012). Interference competition (i.e., predators working against each other to catch food) can decrease the mortality rate in prey relative to what would be expected in an additive model (Soluk & Collins, 1988). Cooperative predation (i.e., predators working together to catch more prey than individuals can by themselves) can lead to higher predation rates than expected (Stallings & Dingeldein, 2012). Both predators and prey can exhibit individual variation that may influence rates of capture (Biro, Abrahams, Post, & Parkinson, 2004; Pruitt et al., 2017). Most predation experiments documenting prey mortality rates in the presence of a single predator species have been done with a simple design of the presence or absence of predators (Clemente, Hernández, Montaño‐Moctezuma, Russell, & Ebert, 2013; Huang, Zheng, Wu, Liu, & Deng, 2016; Kotterba, Kuehn, Hammer, & Polte, 2014; Krueger, Shepherd, & Muir, 2014; Pinto Duarte, Krueger, & Ribeiro, 2013; Sih, Englund, & Wooster, 1998). Notably, some studies have incorporated two predator densities to test for synergy or interference among predators (Griffen, 2006; Griffin, De la Haye, Hawkins, Thompson, & Jenkins, 2008; Ramos & Van Buskirk, 2012; Reiss, Herriot, & Eriksson, 2014; Stier, Geange, & Bolker, 2013; Vance‐Chalcraft, Soluk, & Ozburn, 2004). However, to test for nonlinear effects from predator density on prey mortality requires at least three predator densities in addition to the zero control. Even though the assumption of linear effects of predator density is fundamental to predator–prey interactions, we found only one other paper that used multiple levels of predators to test for linearity in prey mortality rates (Weterings, Umponstira, & Buckley, 2015).

In addition to number of predators, availability of refuge can influence prey mortality rates. Refuge, by definition, should lead to reduced interaction rates between predator and prey (Cressman & Garay, 2009; Sih, 1987). As the amount of available refuge increases, there should be a corresponding decrease in the mortality rate of prey (Savino & Stein, 1982; Westhoff, Watts, & Mattingly, 2013). This decrease in mortality rate should continue as available refuge increases until there is more available refuge than is needed to protect all of the prey in the system at which point mortality rate should plateau (McNair, 1986). Prey may alter their foraging behavior to avoid predators if refuge is available, leading to a lower mortality rate (Luttbeg & Kerby, 2005; Searle, Stokes, & Gordon, 2008). Refuge habitat can decrease mortality rates of prey (Alexander, Kaiser, Weyl, & Dick, 2015; Anderson, 2001; Kovalenko, Dibble, Agostinho, Cantanhêde, & Fugi, 2009; Orrock, Preisser, Grabowski, & Trussell, 2013; Savino & Stein, 1982), and increased number of predators can increase mortality rates of prey, but there have been no clear tests of potential interactions of increasing refuge and increasing predator density.

Most fishes are gape‐limited, and the size of prey relative to gape size of predators can influence mortality rates of prey (Osenberg & Mittelbach, 1989; Scharf, Juanes, & Rountree, 2000). Larger fish can escape gape‐limited predators because they are too big to capture or handle effectively (Yamaguchi & Kishida, 2016). Biomechanically, smaller fish have slower absolute swimming speeds than larger fish (Bainbridge, 1958), thus smaller individuals may not be able to escape from predators as well as larger, faster individuals. Although several studies have focused on effects of body size on mortality rate (Pepin, 2016; Yamaguchi & Kishida, 2016), there are no studies that address the potential interaction between predator density and size of prey, and its effect on mortality rate of prey.

**Figure 1:**
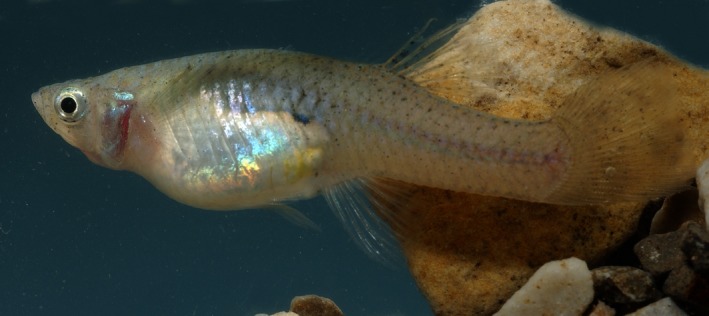
Female western mosquitofish, *Gambusia affinis* (Photograph by C. Riley Nelson)

In this study, we explicitly test for nonlinear patterns in prey mortality resulting from increasing numbers of predators. We use a natural predator–prey system where the predator is gape‐limited (green sunfish, *Lepomis cyanellus*), and prey are size‐structured according to age and gender (western mosquitofish, *Gambusia affinis*; Figure 1) to address two main hypotheses. First, we test to see whether mortality rate of prey is linear and additive with increasing numbers of predators. Second, we test for nonlinear, or nonadditive effects of predator number on prey mortality with increasing levels of habitat availability and variation in body size among prey.

## 2 MATERIALS AND METHODS

To test for nonadditive and interactive effects on prey mortality, we conducted a large, factorial mesocosm experiment and measured mortality rate of western mosquitofish in the presence of varying numbers of green sunfish. We serially ran six blocks of fifteen experimental tanks each. We included predator number (five levels), refuge availability (three levels), and prey size (three levels) as fixed effects in the experiment. We tested predator number by randomly assigning 0, 1, 2, 3, or 4 green sunfish (obtained from a local pond near American Fork, Utah, USA) to each tank within each block. We used a total of 46 green sunfish in the experiment, and they averaged 93 mm standard length (range: 71–113 mm SL). Although we used the same group of predators for the entire experiment, individual predators were randomly reassigned to treatments for each of the six blocks. Thus, the likelihood of any one individual being assigned to the same treatment in multiple blocks was low. Predators that died during the experiment were replaced with predators of similar length. When not being used in a treatment tank, predators were maintained in a common tank of the same size as the treatment tanks and were fed mosquitofish daily.

To test the effects of amount of refuge, three levels of refuge were randomly assigned to each tank within each block. Refuge levels consisted of no refuge, low refuge (20% of available space), and high refuge (80% of available space). We created refuge from cleaned, dead tumbleweeds (*Salsola tragus*), to represent a complex structure similar to common aquatic macrophytes such as *Potamogetan* sp. (Dibble & Thomaz, 2006). We did not use live aquatic plants because the experimental tanks contained no permanent soil substrate, and randomly assigning refuge levels among tanks within each block and assuring uniformity among treatments would have been difficult to accomplish. Tumbleweeds provided a uniform and resilient structure that could be easily moved among tanks as needed, similar to artificial refuge habitat provided by plastic or frayed rope in other studies (Alexander et al., 2015; Laidlaw, Condon, & Belk, 2014; Westhoff et al., 2013). Each tumbleweed was approximately spherical and 45–60 cm in diameter, and we submerged the tumbleweeds in the center of each tank. The low refuge tanks contained one tumbleweed and the high refuge tanks contained four tumbleweeds.

Western mosquitofish to be used as prey in experiments were obtained from ponds in Benjamin, UT. To create three equally available size classes of prey, we introduced 15 fish from three different size classes (juveniles, adult males, and adult females) simultaneously into each tank, for a total of 45 prey fish per tank. We felt it important to have all size classes available to the predator simultaneously to quantify prey mortality rates. However, because we combined the three size classes of prey into one tank, the total number of tanks required for a complete trial was 15 (five predator densities times three refuge levels). We adjusted for the nonindependence of prey size classes within a tank by including tank as a random effect (please find details below). Adult female western mosquitofish averaged 34.4 mm SL (*SD* = 2.1); adult male western mosquitofish averaged 23.3 mm SL (*SD* = 1.9); and juvenile western mosquitofish averaged 21.5 mm SL (*SD* = 1.9). We are aware of the fact that size classes as used here are possibly attended by gender differences in behavior and other traits. We focused on size as a determinant of vulnerability to predation, but some of the variation observed among groups could be attributed to nonsize differences. We filled fifteen 1,100‐L outdoor tanks with approximately 750 L of water and allowed the water to warm to ambient temperatures (temperatures ranged from 17 to 21°C over the course of the experiment) before fish were introduced. We covered the tanks with screens to protect the fish from extrinsic predation. All tanks were aerated continuously during the experiment.

We introduced western mosquitofish into the tanks to acclimate for an hour before predators were introduced into the tank. Predators were not fed for 24 hr prior to being randomly assigned to a treatment tank to assure common levels of hunger. After predators were introduced, we monitored the tanks for 48 hr. On the second day of the experiment, we recorded and replaced any mosquitofish that died from causes other than predation (4.4%). We replaced green sunfish that died as soon as they were noticed (<10%). After 48 hr (enough time for predation to occur while ensuring survival of some of the prey), we captured all remaining fish by draining tanks through a fine mesh net and recorded number of mosquitofish remaining in each size class. We completed six replicate sets of 15 tanks each for a total of 90 trials in the entire experiment.

We scored each individual western mosquitofish as having survived or not, and we analyzed the response with a generalized linear model with mixed effects (GLIMMIX procedure, SAS version 9.2, SAS Institute Inc., Cary, NC). We modeled the response as a binomial function with a logit link function. We considered predator number (five levels, 0–4), refuge amount (three levels), and size class of prey (three levels) as main effects (fixed), and we included all two‐way and three‐way interactions. We considered blocks as random effects to account for the serial nature of the trials and the use of the same group of predators in all blocks (although predators were re‐randomized among blocks). Within each block, we included tank as a random effect because we tested all three size classes of western mosquitofish in each tank. By including tank as a random effect, we adjusted for the nonindependence of size classes of mosquitofish within tanks. Significant interaction terms would indicate nonadditive effects of main effects on prey mortality rate.

## 3 RESULTS

**Figure 2:**
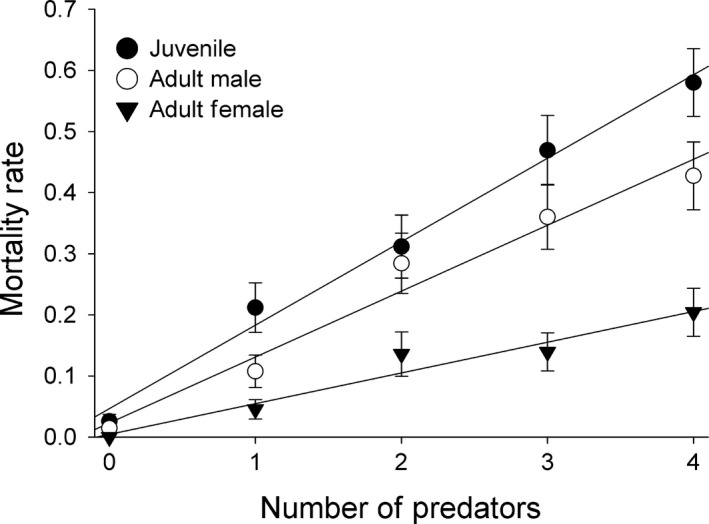
Plot of mean mortality rate of prey (±1 *SE*) by number of predators for each size class of prey. None of the means differ significantly from the linear additive lines of best fit shown, indicating no deviation from a linear additive pattern

Both predator number and the size class of prey significantly affected the mortality rate of prey. Availability of refuge had no significant effect on mortality rate of prey, and there were no significant interactions among main effects (Table 1). Overall, for every predator added, the mortality rate of all prey sizes combined increased linearly by about 10%. Juveniles had the highest mortality rate, followed by adult males and then adult females. For every predator added, juvenile mortality increased by 14%, adult male mortality increased by 11%, and female mortality increased by 5% (Figure 2). Predator number had a linear effect on mortality of the prey population in each size class (Figure 2).

**Table 1:** Effects of predator number, refuge availability, and size class of prey on prey mortality rates from a large mesocosm experiment

Effect	Num DF	Den DF	*F* Value	*p* > *F*
Predator	4	3,828	27.33	**<.0001**
Refuge	2	3,828	0.00	.9999
Size	2	3,828	5.55	**.0039**
Pred × size	8	3,828	0.97	.4559
Pred × refuge	8	3,828	1.57	.1271
Refuge × size	4	3,828	0.57	.6824
Pred × size × refuge	16	3,828	1.21	.2523

All two‐way and three‐way interactions were included. Predators were green sunfish, and prey were western mosquitofish. Bolded *p*‐values represent significant effects.

## 4 DISCUSSION

We found no evidence of nonlinearity in prey mortality rates with increasing predator number. In contrast, previous studies have suggested that nonlinear responses might result from either synergistic or antagonistic interactions as the density of predators increase (Delong & Vasseur, 2013; Stallings & Dingeldein, 2012). There may be several reasons that nonlinear effects are not observed in this study. First, if predators do not interact behaviorally, then antagonistic or synergistic effects will not occur (Pruitt et al., 2017). For example, territorial behavior may limit social interactions among predators (McGregor, 1993), thus precluding antagonistic interactions. Second, antagonistic or synergistic effects among predators may not translate into changes in mortality rates of prey. Antagonistic interactions among individual predators may occur, but foraging time may be increased to offset the difference in prey capture rates. For example, shore crabs were found to engage in antagonistic interactions when multiple conspecifics were present, but there was a corresponding large increase in foraging time compared to when only a single shore crab was present (Whitton, Jenkins, Richardson, & Hiddink, 2012). This increase in foraging time may lead to a similar mortality rate in the prey species for a given number of predators, even though antagonistic behavioral interactions occur. Similarly, for synergistic or cooperative interactions, satiation may limit number of prey consumed such that overall mortality rate of prey may be unchanged. Third, prey exhibit a wide range of antipredator behaviors in response to changes in predator density (Preisser, Orrock, & Oswald, 2007; Smith & Belk, 2001; Stier et al., 2013; Willems & Hill, 2009). Thus, individual level responses by prey may not translate into population level differences in mortality rate because of adjustments in predator foraging time (Loflen & Hovel, 2010) or tradeoffs in prey between feeding and refuging behaviors (Gilliam & Fraser, 1987; Stankowich & Blumstein, 2005).

In a study using odonate naiads as predators and mosquito larvae as prey, Weterings et al. (2015) found that the total number of prey consumed increased with predator density in a nonlinear fashion such that higher densities of predators consumed fewer prey than predicted by a linear relationship. This nonlinear effect is inconsistent with results from our study. However, these results may have occurred because of limited prey numbers relative to the number of predators. In most replicates where predators were at the highest density (i.e., five predators per container), predators consumed 70%–100% of prey (Weterings et al., 2015, figure 4C). Naturally, as prey are depleted total number of prey consumed will plateau. In our experiment, predators, even at the highest density (i.e., four predators per tank) only consumed about 50% of available prey. This difference in relative density of prey may account for the different results obtained between our study and that of Weterings et al. (2015). Additional studies in various predator prey systems are warranted to determine the generality of our results.

We found no evidence of nonlinear effects on prey mortality among different size classes of prey. Size of prey has been shown to have significant effects on the predation rates in gape‐limited predator systems (Osenberg & Mittelbach, 1989; Yamaguchi & Kishida, 2016). Our findings are consistent with general predictions—the largest size class has the lowest mortality and the smallest size class has the highest mortality (Hambright, 1991; Yamaguchi & Kishida, 2016). Even when all size classes are within the gape limitation of the predator, there is preferential selection for prey that minimize handling time per unit weight of prey (Hoyle & Keast, 1987, 1988). In other words, predators try to maximize the reward for the time spent handling prey. This suggests that larger size classes may have a lower predation rate than smaller size classes because of increased handling times or decreased capture probability, which was consistent with our findings. The three different age and gender groups used to represent our three size classes may differ in ways other than size that may affect their mortality rate. For example, males may be more vulnerable to predation, not only because they are smaller, but because they may exhibit sex‐specific behaviors (Magurran & Seghers, 1994; Tobler, Franssen, & Plath, 2008). Such effects may be evident in our study in that although adult males and juvenile fish were somewhat similar in size, mortality rate in adult males was equidistant between females and juveniles.

It is not clear why refuge availability had no effect on mortality rate in this study. Refuge availability has been shown in other systems to decrease the mortality of prey (Alexander et al., 2015; Anderson, 2001; Savino & Stein, 1982). Typically, increased predation risk leads to an increased use of refuge (Loflen & Hovel, 2010). As predator density increased, we predicted increased use of refuge by the prey, and consequent decreased prey mortality per predator (Forrester & Steele, 2004). Our data suggest that the complex structural environment provided by the submerged tumbleweeds did not function as a refuge. The refuge used in this experiment was chosen to mimic spatial configurations and complexity of natural aquatic plants, and increased structural complexity has been shown to function as a refuge in some systems (Belgrad & Griffen, 2016; Huang et al., 2016). However, in other studies with mosquitofish as prey, the refuge is absolute such that only prey are able to move into and out of the refuge at will (Laidlaw et al., 2014; Winkelman & Aho, 1993). It may be that for mosquitofish and green sunfish, refuge habitat that is accessible to both prey and predator provides little benefit in terms of reduced prey mortality rates compared to absolute refuges. Alternatively, prey may not use refuge habitat even when it is available (e.g., Vance‐Chalcraft et al., 2004).

In this study, we documented linear effects of predator number on prey mortality rates in spite of multiple conditions that were suggested to cause nonlinear effects in prey mortality rates. Neither predator–predator interactions, nor prey traits, nor availability of refuge generated nonlinear effects in prey mortality rates with increasing predator number. If our results can be generalized, it appears that predator–prey interactions at the individual level may not scale up to create nonlinear prey mortality rates at the population level. However, controlled experiments conducted at small spatial and temporal scales may not provide adequate complexity to predict population level dynamics at larger spatial and temporal scales. Additional work is required to resolve the effects of predator density on prey mortality rates at larger scales.

## CONFLICT OF INTEREST

None declared.

## 5 AUTHOR CONTRIBUTIONS

RMS participated in interpretation of data and drafting and revising article. MCB participated in design and completion of experiment, analysis of data, interpretation of data, and drafting and revisions of article.
